# Preexisting diabetes and gestational diabetes as risk factors for autism spectrum disorder: an umbrella review

**DOI:** 10.3389/fpubh.2026.1833825

**Published:** 2026-09-09

**Authors:** Nagwa Gad, Hyder O. Mirghani, Abdalla Ali Abdalla, Asmaa Ghmaird, Razaz Baattiah, Mai T. Sindi, Manal Albalawi

**Affiliations:** 1 Department of Pediatrics, Faculty of Medicine, University of Tabuk, Tabuk, Saudi Arabia; 2 Department of Internal Medicine, Faculty of Medicine, University of Tabuk, Tabuk, Saudi Arabia; 3 Department of Pediatrics, Faculty of Medicine, King Abdulaziz University, Jeddah, Saudi Arabia

**Keywords:** autism, gestational diabetes, preexisting diabetes, type 1 diabetes, type 2 diabetes

## Abstract

**Background:**

Maternal diabetes during pregnancy, including preexisting type 1 diabetes (T1DM), type 2 diabetes (T2DM), and gestational diabetes mellitus (GDM), has been investigated as a potential risk factor for autism spectrum disorder (ASD) in offspring. However, the consistency and strength of evidence across published meta-analyses remain uncertain. This umbrella review aimed to synthesize and critically appraise the existing meta-analytic evidence on this association.

**Methods:**

Following the PRISMA 2020 guidelines, we conducted an umbrella review of meta-analyses identified through PubMed, Web of Science, and Google Scholar from inception to March 31, 2026. Eligible studies quantitatively assessed the association between maternal diabetes and the risk of ASD. Of 636 records identified, 11 meta-analyses met the inclusion criteria. Data on effect estimates, heterogeneity, and potential bias were extracted. Study quality was assessed using AMSTAR 2, and overlap among primary studies was evaluated using the corrected covered area (CCA). Given the substantial overlap and methodological variability, the most robust meta-analysis for each exposure was prioritized, and findings were synthesized descriptively without conducting a *de novo* meta-analysis.

**Results:**

Across included meta-analyses, maternal diabetes was consistently associated with increased ASD risk in offspring. Preexisting diabetes showed a more consistent and stronger association compared with GDM, which also demonstrated a positive but more variable association. Heterogeneity was commonly moderate to high, and publication bias was variably reported. Overall evidence ranged from suggestive to weak, with higher credibility observed for preexisting diabetes than for GDM.

**Conclusion:**

A consistent and statistically significant association was observed between maternal diabetes—including both preexisting diabetes and GDM—and ASD, with evidence suggesting a risk gradient according to diabetes type. These findings highlight the importance of maternal metabolic health during pregnancy and support further research into the underlying mechanisms and potential preventive strategies.

**Systematic review registration:**

https://www.crd.york.ac.uk/PROSPERO/view/CRD420261334307, 2026CRD420261334307.

## Introduction

ASD is a serious neurodevelopmental disorder characterized by impairments in social communication and interaction. The severity of ASD varies considerably among affected individuals. Diagnosis is typically established during the first 3 years of age. The reported prevalence of ASD ranges from 0.4% in Asia to 1.7% in Australia (1). Cognitive flexibility deficits, particularly perseverative errors, are common co-occurring features among individuals with ASD (2).

Previous studies have identified structural and functional brain alterations in both ASD and attention-deficit/hyperactivity disorder (ADHD), although the patterns differ between the two conditions. Individuals with ASD tend to exhibit increased gray matter volume in the frontotemporal regions, whereas those with ADHD often show reduced gray matter volume in the orbitofrontal cortex (3). In addition, low vitamin D levels, along with elevated folate and vitamin B12 levels, have been associated with ASD (4). However, evidence regarding folate levels remains inconsistent. A systematic review and meta-analysis reported that folic acid supplementation of at least 400 μg/day was associated with a reduced risk of ASD.

Although the etiology of ASD remains elusive, it is thought to result from interactions between genetic susceptibility and environmental factors. Because ASD is typically diagnosed before 3 years of age, prenatal factors are considered particularly important (5). Prenatal exposures, including infections, antibiotics, antidepressant medications, environmental toxins, and maternal hyperglycemia during pregnancy, have all been implicated as potential risk factors. Proposed underlying mechanisms include hormonal dysregulation, gut microbiota alterations, oxidative stress, immune dysfunction, and mitochondrial dysfunction (6).

Among environmental exposures, lead exposure has been associated not only with ASD but also with hearing impairment and age-related visual disorders (7). Other factors associated with an increased risk of ASD include preterm birth and low birth weight. In contrast, GDM is typically associated with fetal overgrowth and large-for-gestational-age births due to maternal hyperglycemia and subsequent fetal hyperinsulinemia (8). However, pregnancies complicated by GDM are also at increased risk of adverse obstetric outcomes, including placental dysfunction and preterm delivery. These complications may result from maternal metabolic dysregulation, systemic inflammation, or vascular impairment, potentially leading to medically indicated early delivery or impaired fetal growth (9).

GDM, a metabolic disorder with increasing global prevalence, is a form of diabetes diagnosed during pregnancy, typically emerging in the second or third trimester as insulin resistance increases. Uncontrolled maternal blood glucose levels may adversely affect offspring neurodevelopment (10). In contrast, preexisting diabetes includes T1DM, an autoimmune disorder characterized by pancreatic β-cell destruction and absolute insulin deficiency, and T2DM, which is primarily characterized by insulin resistance and relative insulin deficiency. These conditions differ in pathophysiology, timing of onset, and severity of maternal hyperglycemia, potentially resulting in varying degrees and durations of fetal exposure to metabolic dysregulation and, consequently, different impacts on offspring neurodevelopment (11).

Evidence from both human and animal studies has linked maternal diabetes to motor and cognitive impairments, ASD, ADHD, learning difficulties, and other psychiatric disorders in offspring (12).

According to the International Diabetes Federation (2021), approximately 21.1 million live births worldwide, representing 16.7% of all births, are affected by some form of hyperglycemia during pregnancy. This high prevalence represents a significant global health concern, particularly in low- and middle-income countries, where limited access to adequate obstetric care and early screening may increase the risk of complications for both mothers and their infants (13).

Several systematic reviews and meta-analyses have examined the association between maternal diabetes and ASD, but their findings vary in scope, exposure definitions, and methodological quality. Moreover, substantial overlap exists among the included primary studies, making interpretation challenging. An umbrella review is therefore needed to critically appraise, compare, and synthesize the existing meta-analytic evidence, assess methodological quality, evaluate overlap, and determine the overall credibility of the association between maternal diabetes sub-types and ASD risk.

This umbrella review aimed to assess the relationship between GDM and preexisting diabetes and ASD.

## Subjects and methods

This umbrella review was conducted in accordance with the Preferred Reporting Items for Systematic Reviews and Meta-Analyses (PRISMA 2020) guidelines, alongside methodological recommendations specific to umbrella reviews. The aim was to systematically appraise and synthesize evidence from published meta-analyses examining the association between maternal diabetes (preexisting T1DM, preexisting T2DM, and GDM) and the risk of ASD in offspring. The protocol was registered in PROSPERO. PROSPERO 2026 CRD420261334307. Available from https://www.crd.york.ac.uk/PROSPERO/view/CRD420261334307.

### Research question (PICOS framework)

Population (P): Pregnant women and their offspring.
Exposure (I): Maternal preexisting T1DM, preexisting T2DM, and GDM.
Comparator (C): Pregnant women without diabetes.
Outcome (O): ASD in offspring.
Study design (S): Systematic reviews and meta-analyses of observational studies.

### Eligibility criteria

#### Inclusion criteria

We included systematic reviews with meta-analyses that quantitatively evaluated the association between maternal diabetes (T1DM, T2DM, or GDM) and ASD risk, reporting pooled effect estimates odds ratios (ORs), risk ratios (RRs), hazard ratios (HRs), or standardized mean differences with corresponding 95% confidence intervals.

#### Exclusion criteria

Primary studies (cohort, case–control, cross-sectional), randomized trials, narrative reviews, editorials, letters, and studies not reporting relevant exposure–outcome associations were excluded.

### Literature search

**Figure 1:**
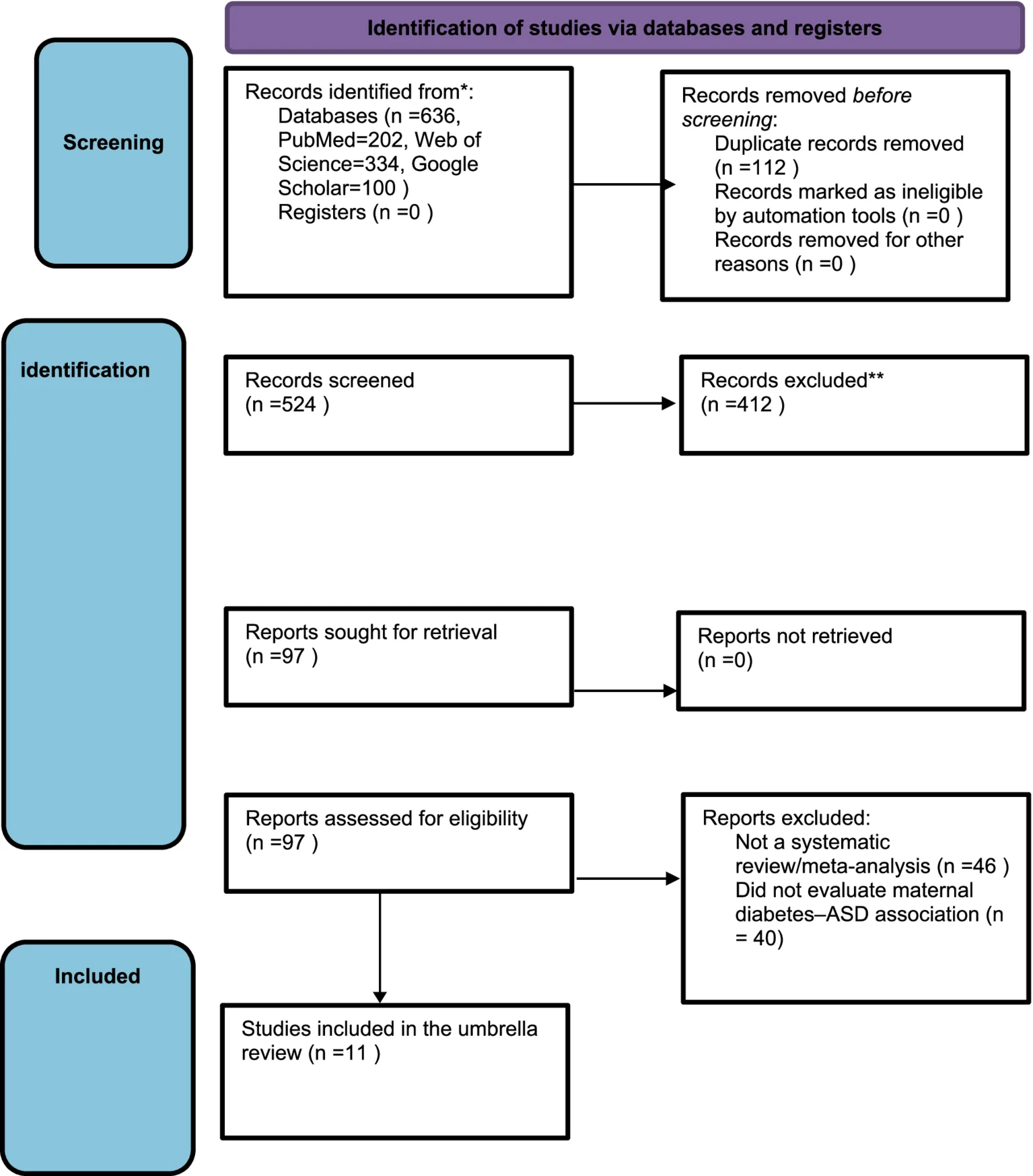
PRISMA 2020 flow diagram of the study selection process for the umbrella review evaluating the association between maternal diabetes and autism spectrum disorder (ASD).

Two researchers (N.G. and H.M.) independently conducted a comprehensive literature search in PubMed/MEDLINE, Web of Science, and Google Scholar from database inception to March 31, 2026. Search strategies combined controlled vocabulary and free-text terms related to ASD and maternal diabetes using Boolean operators. The PubMed search strategy included: (“autism spectrum disorder” OR ASD OR autism) AND (“gestational diabetes” OR GDM OR “type 1 diabetes” OR T1DM OR “type 2 diabetes” OR T2DM OR “maternal diabetes”) AND (“systematic review” OR “meta-analysis”). The Web of Science search strategy was: TS = (“autism spectrum disorder” OR ASD OR autism) AND TS = (“gestational diabetes” OR GDM OR “type 1 diabetes” OR T1DM OR “type 2 diabetes” OR T2DM OR “maternal diabetes”) AND TS = (“systematic review” OR “meta-analysis”). The Google Scholar search strategy was: autism AND (“gestational diabetes” OR “type 1 diabetes” OR “type 2 diabetes” OR “maternal diabetes”) AND (“systematic review” OR “meta-analysis”). Google Scholar was used as a supplementary source to identify potentially missed studies or gray literature, with the first 100 results screened due to reproducibility limitations. A total of 636 records were identified through database searching (PubMed/MEDLINE = 202; Web of Science = 334; Google Scholar = 100). After removal of 112 duplicates, 524 records underwent title and abstract screening, of which 427 were excluded. The full texts of 97 systematic reviews were assessed for eligibility, and 86 were excluded because they were either not systematic reviews or did not report relevant outcomes (Figure 1).

### Data extraction

Data were extracted from the included meta-analyses using a standardized form. Extracted variables included author, year of publication, number of included studies, population characteristics, exposure definitions (T1DM, T2DM, and GDM), ASD outcome ascertainment, pooled effect estimates (OR, RR, HR) with 95% confidence intervals, and degree of covariate adjustment. Definitions of exposures and outcomes were recorded as reported in the original meta-analyses; no harmonization was performed. The extracted data are presented in Tables 1, 2.

**Table 1:** Summary of pooled effect estimates from the included meta-analyses evaluating the association between maternal diabetes and autism spectrum disorder (ASD).

Study	Exposure	Effect measure	Effect size	95% CI (Lower)	95% CI (Upper)
Gestational diabetes mellitus (GDM)					
Garza-Martínez et al., 2025 (17)	GDM	OR	1.31	1.16	1.47
Guan et al., 2025 (18)	GDM	OR	1.38	1.20	1.59
Guo et al., 2025 (19)	GDM	RR*	1.30	1.16	1.46
Maryam et al., 2025 (20)	GDM	OR	1.62	1.44	1.82
Rowland et al., 2021 (21)	GDM	OR	1.42	1.22	1.65
Ye et al., 2025 (26)	Maternal diabetes (GDM included)	RR	1.25	1.20	1.31
Type 2 diabetes mellitus (T2DM)					
Garza-Martínez et al., 2025 (17)	T2DM	OR	1.48	1.31	1.68
Type 1 diabetes mellitus (T1DM)					
Garza-Martínez et al., 2025 (17)	T1DM	OR	1.73	1.05	2.87
Yamamoto et al., 2019 (25)	Preexisting diabetes	RR	1.98	1.46	2.68
Any maternal diabetes					
Wan et al., 2018 (22)	Maternal diabetes	RR	1.48	1.26	1.75
Wang et al., 2017 (23)	Maternal diabetes	RR	1.49	1.18	1.89
Xu et al., 2014 (24)	Maternal diabetes	RR	1.48	1.25	1.75

ASD, autism spectrum disorder; OR, odds ratio; RR, risk ratio; CI, confidence interval.

*Effect estimates are reported as presented in the original meta-analyses; no statistical conversion between OR and RR was performed.

†Studies are grouped by exposure type for clarity; this categorization was not applied in the original meta-analyses.

**Table 2:** Summary of exposure ascertainment, outcome definition, and confounding adjustment across included meta-analyses.

Study	Exposure ascertainment	ASD outcome definition	Key covariates adjusted for
Bravo-Muñoz et al., 2025 (16)	Clinical and registry data	DSM/registry codes	Age, BMI, SES, comorbidities
Garza-Martínez et al., 2025 (17)	Registry/hospital ICD codes	DSM/ICD/clinical assessment	Age, BMI, smoking, SES
Guan et al., 2025 (18)	OGTT-based clinical criteria	DSM/ICD codes	Age, BMI, birth factors
Guo et al., 2025 (19)	Medical records	DSM/ICD	Age, BMI, SES
Maryam et al., 2025 (20)	OGTTand clinical guidelines	Registry/clinical diagnosis	Age, BMI, smoking
Rowland et al., 2021 (21)	Clinical diagnostic criteria	DSM-IV/DSM-5/ICD	Age, BMI, SES, smoking
Wan et al., 2018 (22)	Registry/medical records	DSM/ICD	Age, gestational factors
Wang et al., 2017 (23)	Registry-based	DSM-IV/DSM-5	Age, SES, obstetric factors
Xu et al., 2014 (24)	Self-report + records	DSM/ICD	Age, SES, parity
Yamamoto et al., 2019 (25)	Hospital + registry	ICD codes	SES, obstetric factors
Ye et al., 2025 (26)	ICD-based databases	DSM/ICD/registry	Age, BMI, SES, smoking

SES, socioeconomic status; DSM, Diagnostic and Statistical Manual of Mental Disorders; ICD, International Classification of Diseases.

### Quality assessment

The methodological quality of the included meta-analyses was evaluated using the AMSTAR 2 (A Measurement Tool to Assess Systematic Reviews) instrument (14). The results of this assessment are summarized in Table 3.

**Table 3:** AMSTAR-2 quality assessment of the included meta-analyses.

Author	Protocol registered	Adequacy of literature search	Risk of bias assessment	publication bias assessment reported	Overall AMSTAR-2 quality rating
Bravo-Muñoz et al., 2025 (16)	Yes	Yes	Yes	Yes	High
Garza-Martínez et al., 2025 (17)	Yes	Yes	Yes	Yes	High
Guan et al., 2025 (18)	Yes	Yes	Yes	Yes	High
Guo et al., 2025 (19)	Yes	Yes	Yes	Yes	Moderate
Maryam et al., 2025 (20)	No	Yes	Partial	Yes	Low
Rowland et al., 2021 (21)	No	Yes	Partial	Yes	Low
Wan et al., 2018 (22)	No	Partial	No	Partial	Low
Wang et al., 2017 (23)	Yes	Yes	Yes	Yes	Moderate
Xu et al., 2014 (24)	Yes	Yes	Yes	Yes	High
Yamamoto et al., 2019 (25)	Yes	Yes	Yes	Yes	Moderate
Ye et al., 2025 (26)	Yes	Yes	Yes	Yes	High

AMSTAR-2 domains are reported as: Yes = fully met; Partial Yes = partially met or unclear reporting; No = not met.

### Assessment of overlap

Overlap of primary studies across the included meta-analyses was assessed by identifying shared studies and calculating the Corrected Covered Area (CCA) (15). Across the 11 meta-analyses, 189 study entries were identified, of which 169 were duplicates, yielding 20 unique studies. The resulting CCA was 84.5%, indicating very high overlap. To address substantial overlap (CCA = 84.5%), we prioritized one representative meta-analysis for each exposure–outcome association using predefined criteria applied hierarchically: (1) highest AMSTAR 2 methodological rating, (2) most recent publication date; (3) largest number of included primary studies, (4) greater exposure specificity (separate estimates for T1DM, T2DM, and GDM), and (5) more comprehensive adjustment for confounding variables. Other overlapping reviews were retained for qualitative comparison (Table 4).

**Table 4:** Evidence credibility for the association between maternal diabetes and autism spectrum disorder (ASD).

Study	Exposure	Outcome	Effect size (95% CI)	Evidence level
Bravo-Muñoz et al., 2025 (16)	GDM	ASD	RR 1.23 (1.13–1.34)	Suggestive
Garza-Martínez et al., 2025 (17)	Type 2 diabetes mellitus (T2DM)	ASD	OR 1.48 (1.31–1.68)	Suggestive
Garza-Martínez et al., 2025 (17)	Type 1 diabetes mellitus (T1DM)	ASD	OR 1.73 (1.05–2.87)	Weak
Garza-Martínez et al., 2025 (17)	GDM	ASD	OR 1.31 (1.16–1.47)	Suggestive
Guan et al., 2025 (18)	GDM	ASD	OR 1.38 (1.20–1.59)	Suggestive
Guo et al., 2025 (19)	GDM	ASD	RR 1.30 (1.16–1.46)	Suggestive
Maryam et al., 2025 (20)	GDM	ASD	OR 1.62 (1.44–1.82)	Suggestive
Rowland et al., 2021 (21)	GDM	ASD	OR 1.42 (1.22–1.65)	Suggestive
Wan et al., 2018 (22)	Maternal diabetes	ASD	RR 1.48 (1.26–1.75)	Suggestive
Wang et al., 2017 (23)	Maternal diabetes	ASD	RR 1.49 (1.18–1.89)	Suggestive
Xu et al., 2014 (24)	Maternal diabetes	ASD	RR 1.48 (1.25–1.75)	Suggestive
Yamamoto et al., 2019 (25)	Preexisting diabetes	ASD	RR 1.98 (1.46–2.68)	Suggestive
Ye et al., 2025 (26)	Maternal diabetes	ASD	RR 1.25 (1.20–1.31)	Highly suggestive

ASD, autism spectrum disorder; OR, odds ratio; RR, risk ratio; CI, confidence interval; GDM, gestational diabetes mellitus.

Evidence levels were assigned based on effect size, consistency, sample size, and risk of bias across the included meta-analyses.

### Assessment of bias and evidence credibility

Publication bias, small-study effects, and heterogeneity (I^2^) were summarized as reported in the included meta-analyses. Where available, results of Egger’s test and funnel plot assessments were recorded and interpreted cautiously. Evidence credibility was graded using umbrella review criteria adapted from established evidence-classification frameworks. Associations were categorized as: Highly suggestive: (*p* < 0.001, large sample size, consistent direction of effect, moderate or low heterogeneity (I^2^ < 50%), no major risk-of-bias concerns, and no strong evidence of small-study effects), suggestive: statistically significant association (*p* < 0.05, moderate-to-large sample size, generally consistent findings, but with some heterogeneity or methodological limitations), and weak: (nominal statistical significance (*p* < 0.05) but limited by small sample size, wide confidence intervals, substantial heterogeneity, or methodological concerns).

The evidence was downgraded when substantial overlap (high CCA), high risk of bias, or publication bias was identified. The results are presented in Tables 4, 5.

**Table 5:** Summary and grading of evidence for the association between maternal diabetes and autism spectrum disorder (ASD).

Exposure	Outcome	No. of meta-analyses	Representative effect estimate (range of 95% CI)*	Range of pooled effect estimates	Heterogeneity (I^2^ range)	AMSTAR 2 (modal rating)**	Small-study effects / publication biass***	Overlap (CCA)	Evidence level****	Interpretation
Any maternal diabetes	ASD	11	RR 1.25–1.49	1.23–1.98	0–82%	Moderate–high	Inconsistent	Very high (84.5%)	Highly suggestive	Consistent positive association across meta-analyses; interpretation limited by substantial heterogeneity and very high overlap of primary studies
Gestational diabetes (GDM)	ASD	7	OR 1.31–1.43	1.23–1.62	30–82%	Moderate	Possible	Very high (84.5%)	Suggestive	Modest but consistent association; heterogeneity likely reflects differences in diagnostic criteria, confounder adjustment, and study design across studies.
Preexisting diabetes (T1DM/T2DM)	ASD	4	OR 1.48–1.73	1.36–1.98	10–55%	High	Limited evidence of bias.	Very high (84.5%)	Highly suggestive	Slightly stronger and more consistent association than GDM; interpretation is influenced by overlapping evidence and potential residual confounding.

* Representative effect estimate reflects results from the most comprehensive and methodologically robust meta-analyses (based on sample size, recency, and AMSTAR 2 rating). Effect measures (RR, OR) are reported as originally presented; no transformation was performed.

** Modal rating reflects the most frequent rating across included meta-analyses.

*** Based on reported assessments (e.g., Egger’s test, funnel plot asymmetry), categorized as inconsistent, possible, or limited evidence.

**** Evidence levels were assigned using umbrella review criteria, considering statistical significance, sample size, consistency, heterogeneity, risk of bias, and overlap.

### Data synthesis

No *de novo* meta-analysis or re-pooling of effect sizes was performed. Effect estimates were extracted and reported as presented in the original meta-analyses. Findings were synthesized descriptively, with emphasis on the direction and magnitude of associations, consistency across studies, and methodological quality.

Given the substantial overlap of primary studies and variability in effect measures (OR, RR, and HR), quantitative aggregation across meta-analyses was not undertaken.

### Statistical analysis

This umbrella review did not involve *de novo* quantitative synthesis or re-analysis of primary study data. No pooled analyses or new summary effect estimates were generated using statistical software. Instead, effect estimates (OR, RR, HR) were extracted as reported in the included meta-analyses and presented descriptively. For associations evaluated by multiple meta-analyses, the most comprehensive and methodologically robust study (based on sample size, recency, and AMSTAR 2 rating) was prioritized, while others were used for qualitative comparison.

Heterogeneity (I^2^), publication bias (e.g., Egger’s test), and small-study effects were summarized as reported in the original meta-analyses. No additional funnel plots or statistical tests were performed. The strength and credibility of evidence were assessed using established umbrella review criteria, incorporating statistical significance, sample size, consistency, heterogeneity, and risk of bias.

Egger’s and Begg’s tests were interpreted as reported in the original meta-analyses. Funnel plot assessments were based on visual inspection where applicable.

## Results

A total of 11 meta-analyses (15–26) were included, evaluating the association between maternal diabetes (preexisting T1DM, preexisting T2DM, and gestational diabetes mellitus [GDM]) and the risk of autism spectrum disorder (ASD) in offspring. No new quantitative synthesis was performed, and findings are reported as extracted from the original meta-analyses.

### Summary of effect estimates

The pooled effect estimates reported by the included meta-analyses are summarized in Table 1. Across all studies, maternal diabetes exposures were consistently associated with an increased risk of ASD in offspring. Reported effect sizes ranged from 1.23 to 1.98, indicating a modest to moderate increase in risk.

### Gestational diabetes mellitus (GDM)

GDM was the most frequently examined exposure, with effect estimates generally ranging from 1.23 to 1.62 across meta-analyses (e.g., Bravo-Muñoz et al., RR = 1.23 [1.13–1.34]; Maryam et al., OR = 1.62 [1.44–1.82]). Other studies (e.g., Guan et al., Guo et al., Rowland et al.) reported similar positive associations within a comparable range.

### Preexisting diabetes

Preexisting diabetes (T1DM and T2DM) was evaluated in fewer meta-analyses but generally showed somewhat higher effect estimates in some cases (e.g., Garza-Martínez et al.: T1DM OR = 1.73 [1.05–2.87], T2DM OR = 1.48 [1.31–1.68]; Yamamoto et al.: RR = 1.98 [1.46–2.68]). However, these estimates were based on a smaller evidence base and, in some cases, wider confidence intervals. Meta-analyses assessing overall maternal diabetes (without distinguishing type) also reported consistent positive associations, with effect estimates typically ranging from 1.25 to 1.49 (e.g., Wan et al., Wang et al., Xu et al., Ye et al.).

Despite differences in effect measures (OR, RR), populations, and exposure definitions, the direction of association was consistently positive across all included meta-analyses.

### Consistency and heterogeneity across Meta-analyses

Several included meta-analyses reported moderate to high heterogeneity (I^2^ up to 82%), likely reflecting differences in study populations, diagnostic criteria for GDM and ASD, and adjustment for confounding variables. In contrast, some meta-analyses, particularly those evaluating preexisting diabetes, reported low heterogeneity, suggesting more consistent findings within these subgroups.

### Overlap across meta-analyses

A very high degree of overlap in primary studies was identified (Corrected Covered Area = 84.5%), with only 20 unique studies represented across the 11 meta-analyses. This substantial redundancy indicates that many meta-analyses were based on largely overlapping datasets, which may inflate the apparent consistency and precision of the observed associations. Accordingly, greater emphasis was placed on the most comprehensive and methodologically robust meta-analyses, while others were used for qualitative comparison.

### Quality assessment and risk of bias

The methodological quality of the included meta-analyses, assessed using AMSTAR 2, ranged from moderate to low (Table 3), with common limitations including incomplete reporting of search strategies and insufficient assessment of risk of bias.

Publication bias and small-study effects were inconsistently evaluated across studies. Where reported, some meta-analyses suggested potential small-study effects; however, findings were not consistent and should be interpreted cautiously given heterogeneity and overlap.

### Credibility and strength of evidence

Based on umbrella review criteria incorporating statistical significance, sample size, heterogeneity, overlap, and risk of bias, the overall strength of evidence for the association between maternal diabetes and ASD was classified as suggestive to highly suggestive, but not convincing. This grading reflects the consistent direction of association across studies, but also limitations related to heterogeneity, potential bias, and the very high overlap of primary studies (Tables 4, 5).

Publication bias and small-study effects are shown in Table 6.

**Table 6:** Publication bias and small-study effects in included meta-analyses.

Meta-analyses	Bias assessed	Method used	Publication bias and small-study effects
Bravo-Muñoz et al., 2025 (16)	Yes	Egger’s test + Funnel plot analysis	No significant publication bias
Garza-Martínez et al., 2025 (17)	Yes	Begg’s test + Egger’s test	Possible asymmetry
Guan et al., 2025 (18)	Yes	Funnel plot analysis	No major bias
Guo et al., 2025 (19)	Yes	Egger’s test	No evidence of publication bias
Maryam et al., 2025 (20)	Yes	Funnel plot analysis	Possible bias
Rowland et al., 2021 (21)	Yes	Egger’s test	Inconclusive evidence of publication bias
Wan et al., 2018 (22)	Partially reported	Not clearly reported	Uncertain
Wang et al., 2017 (23)	Yes	Egger’s test	Possible small-study effects
Xu et al., 2014 (24)	Partially reported	Funnel plot analysis	Uncertain
Yamamoto et al., 2019 (25)	Yes	Egger’s test	No significant bias
Ye et al., 2025 (26)	Yes	Egger’s test+ Funnel plot analysis	Minimal evidence of publication bias

## Discussion

In this umbrella review, evidence from the included meta-analyses indicated an association between maternal diabetes during pregnancy and an increased risk of ASD in offspring. Across meta-analyses, any form of maternal diabetes was generally associated with a statistically significant increase in ASD risk.

When stratified by diabetes sub-type, preexisting diabetes (T1DM and T2DM) showed a stronger and more consistent association with ASD across four meta-analyses. Gestational diabetes mellitus (GDM) was also associated with an increased risk of ASD; however, effect estimates showed greater variability across studies.

Overall, the pattern of findings across meta-analyses suggests a relatively consistent positive association between maternal diabetes exposure and ASD risk, with some variation in effect size according to diabetes sub-type.

The association appears slightly stronger for preexisting diabetes, which may reflect exposure during early fetal development, including critical neurodevelopmental windows in the first trimester, as well as a longer duration of maternal hyperglycemia and a greater burden of metabolic and microvascular complications.

From a clinical perspective, this difference may be explained by the timing and duration of fetal exposure to hyperglycemia. Preexisting (pregestational) diabetes may affect early embryonic and fetal neurodevelopmental processes, whereas gestational diabetes mellitus (GDM) is typically diagnosed later in the second or third trimester (27, 28). This temporal difference may partially account for the observed gradient in risk across diabetes sub-types.

Biological mechanisms underlying the association between hyperglycemia during pregnancy and ASD include oxidative stress, systemic inflammation, and altered insulin and insulin-like growth factor signaling, all of which may adversely affect fetal brain development (29, 30). In addition, emerging evidence suggests that epigenetic modifications induced by the intrauterine metabolic environment may contribute to long-term neurodevelopmental outcomes in offspring (24, 31).

Gestational diabetes mellitus may also contribute to ASD risk through maternal inflammation–mediated immune activation. GDM is associated with chronic low-grade inflammation, which may trigger maternal immune activation (MIA) during pregnancy. MIA has been hypothesized to disrupt fetal neurodevelopment through inflammatory signaling pathways, thereby increasing the risk of autism spectrum disorder in offspring (32).

Another important mechanism linking maternal diabetes and ASD risk is co-occurring maternal obesity. Previous studies have shown that maternal obesity and diabetes during pregnancy are associated with higher autism screening scores in toddlers, indicating an increased risk of early autistic traits. Stronger associations have been observed with increasing severity of maternal obesity, while both preexisting diabetes and early-diagnosed gestational diabetes (≤26 weeks) have also been linked to elevated scores. Importantly, these associations persisted even after excluding children later diagnosed with ASD, suggesting that maternal metabolic conditions may contribute to broader atypical neurodevelopment, with obesity potentially acting as a mediating factor in diabetes-related risk (33, 34).

Maternal obesity is also an independent risk factor for ASD. Evidence suggests that obesity, particularly when combined with type 2 diabetes, is associated with increased ASD risk in offspring, potentially mediated by elevated maternal branched-chain amino acid levels. Stronger associations have been reported with higher levels of maternal obesity and with preexisting or early gestational diabetes, further supporting a broader impact on atypical neurodevelopment (13).

Increased inflammation, oxidative stress, hypoxia, and hyperinsulinemia during pregnancy may disrupt fetal brain development and contribute to later neurodevelopmental, cognitive, and behavioral outcomes. Maternal obesity, chronic inflammation, and diabetes may exert combined effects, potentially increasing the risk of ASD beyond that associated with any single condition. In addition, the severity and type of maternal diabetes (e.g., T1DM, T2DM, or insulin-treated GDM) may further influence risk (35, 36).

A recent retrospective study conducted in Isreal and included 115,063 deliveries (3,461 were GDM diet-controlled, and 1,164 required pharmacological treatment). Seven hundred sixty-seven of the offspring developed ASD. No significant statistical difference was found between GDM and ASD after controlling for various confounders, including maternal age at delivery, gestational age at delivery, race, gender, hypertension, birth weight, and 5-min Apgar score (37).

The association between maternal diabetes and ASD is likely influenced by important confounding and interacting factors. Maternal obesity, which commonly co-occurs with diabetes, is independently associated with ASD and may act as both a confounder and a mediator through inflammatory and metabolic pathways. The degree of glycemic control also appears relevant, with more severe hyperglycemia associated with greater neurodevelopmental risk, suggesting a potential dose–response relationship (25). In addition, shared genetic liability may partially explain these findings, as inherited factors related to both metabolic dysfunction and neurodevelopment may increase risk in both mother and child. Genetically informed and sibling comparison studies showing attenuation of associations further support this interpretation, suggesting that the relationship is multi factorial rather than purely causal (38).

This study has several strengths. First, it provides a comprehensive synthesis of existing meta-analyses. Second, it separately evaluates preexisting diabetes and gestational diabetes mellitus (GDM), allowing more detailed exposure stratification. However, several limitations should be acknowledged.

*The study limitations*: The current study has several limitations. All included evidence is derived from observational studies and is therefore subject to residual confounding, including maternal obesity, advanced maternal age, socioeconomic factors, and shared genetic susceptibility, which may partially explain the observed associations (39). Notably, studies using more rigorous designs, such as sibling comparison analyses, have reported attenuation of effect sizes, suggesting that familial and environmental confounding may contribute to the observed relationship (40, 41).

Additional limitations include substantial overlap of primary studies across meta-analyses, heterogeneity in diagnostic criteria for both diabetes and ASD, and limited data on glycemic control, treatment, and timing of exposure.

### Clinical implications

Although the relative increase in ASD risk is statistically significant, the absolute risk increase is likely small. Therefore, these findings should not be interpreted as indicating a substantial individual risk but rather as highlighting maternal metabolic health as one of several contributing factors to neurodevelopmental outcomes.

Optimizing glycemic control before and during pregnancy remains essential, not only for preventing obstetric and neonatal complications but also for potentially supporting favorable long-term neurodevelopmental outcomes in offspring.

## Conclusion

Maternal diabetes, including both preexisting diabetes and gestational diabetes mellitus (GDM), is associated with a modest but consistent increase in the risk of ASD in offspring. While the evidence is statistically significant and biologically plausible, a causal relationship cannot be established due to potential residual confounding and the observational nature of the underlying evidence. Clinically, these findings highlight the importance of optimal glycemic management before and during pregnancy. Future research should focus on clarifying causal mechanisms, the role of glycemic control and timing of exposure, and the potential for targeted preventive strategies.

## Data Availability

The original contributions presented in the study are included in the article/Supplementary material, further inquiries can be directed to the corresponding author.
