# Supplementary material for: Preexisting diabetes and gestational diabetes as risk factors for autism spectrum disorder: an umbrella review
Source: Front Public Health. 2026 Sep 9;14:1833825. doi: 10.3389/fpubh.2026.1833825 (PMC13597867; doi:10.3389/fpubh.2026.1833825)
Supplement: Supplementary file 1 [file Table_1.DOCX]

**Supplementary Table S1. Literature search strategy and results (From inception to March 31, 2026).**

| Database | Search Strategy | Filter applied | 201 |
| --- | --- | --- | --- |
| **PubMed/MEDLINE** | (“autism spectrum disorder”[MeSH] OR ASD OR autism) AND (“gestational diabetes” OR GDM OR “type 1 diabetes” OR T1DM OR “type 2 diabetes” OR T2DM OR “maternal diabetes”) AND (“systematic review” OR “meta-analysis”) | Humans; English; Article types: systematic reviews, meta-analyses |  |
| **Web of Science Core Collection** | TS=(“autism spectrum disorder” OR ASD OR autism) AND TS=(“gestational diabetes” OR GDM OR “type 1 diabetes” OR T1DM OR “type 2 diabetes” OR T2DM OR “maternal diabetes”) AND TS=(“systematic review” OR “meta-analysis”) | Document types: Review; Language: English. | 344 |
| **Google Scholar** | autism AND (“gestational diabetes” OR “type 1 diabetes” OR “type 2 diabetes” OR “maternal diabetes”) AND (“systematic review” OR “meta-analysis” | Sorted by relevance; first 100 results screened | 100 |
